# Efficiently Characterizing the Quantum Information Flow, Loss, and Recovery in the Central Spin System

**DOI:** 10.3390/e26121077

**Published:** 2024-12-10

**Authors:** Jiahui Chen, Mohamad Niknam, David Cory

**Affiliations:** 1 Institute for Quantum Computing, Waterloo, ON N2L 3G1, Canada; dcory@uwaterloo.ca; 2 Department of Physics, University of Waterloo, Waterloo, ON N2L 3G1, Canada; 3 Physics and Astronomy Department, University of California, Los Angeles, CA 90095-1059, USA; mniknam@physics.ucla.edu; 4 Department of Chemistry, University of Waterloo, Waterloo, ON N2L 3G1, Canada

**Keywords:** quantum information flow, qubit characterization, quantum computing, physical qubits, quantum error mitigation, entanglement entropy

## Abstract

Understanding the flow, loss, and recovery of the information between a system and its environment is essential for advancing quantum technologies. The central spin system serves as a useful model for a single qubit, offering valuable insights into how quantum systems can be manipulated and protected from decoherence. This work uses the stimulated echo experiment to track the information flow between the central spin and its environment, providing a direct measure of the sensitivity of system/environment correlations to environmental dynamics. The extent of mixing and the growth of correlations are quantified through autocorrelation functions of the noise and environmental dynamics, which also enable the estimation of nested commutators between the system/environment and environmental Hamiltonians. Complementary decoupling experiments offer a straightforward measure of the strength of the system Hamiltonians. The approach is experimentally demonstrated on a spin system.

## 1. Introduction

Advances in quantum technologies are transforming fields such as communication [1], sensing [2], and physics simulation [3,4], but decoherence remains an obstacle. In particular, the memory of the environment plays a key role in determining the optimal strategies for control [3,5,6,7] and error correction [8], which is not captured by Markovian approximations such as Lindbladians [9]. Here, we present the theory and experimental implementation of a central spin (CS) model (Figure 1) [10,11] of qubit decoherence that characterizes the memory effects in the environment. The CS is a spin-1/2 interacting with an environment that can hold quantum information. The Hamiltonian of the system is

(1) $$H=H_{cs}+H_{cs/e}+H_{e},$$

where $H_{cs}$ and $H_{e}$ are the Hamiltonians of the CS and environment, respectively, and $H_{cs/e}$ describes the coupling of the CS and the environment. Note that there is no dissipation in this system; the total dynamics are unitary and thus perfectly preserve the quantum information. The essence of this model is that an initially separable CS becomes correlated to the environment through the CS/environment coupling [12]. The coherence time of the CS, to first order, is governed by the rate at which these correlations form. The correlations are refocusable unless disrupted by the environment Hamiltonian, leading to a loss of refocusability with a characteristic time, *T*, which is determined by $\left[H_{cs/e},H_{e}\right]$. As higher correlations build, the quantum information shared among larger correlated states becomes more susceptible to $H_{e}$. The loss of refocusability has an initial Zeno region [13], marked by a Gaussian signature in the initial decay of the autocorrelation function, which represents the short-term memory of the environment. This region, where the quantum information is well preserved, is important for the optimal design of quantum gates. Furthermore, the environment may hold part of the quantum information and have a long-term memory, which can be much harder to detect. This long-term memory can lead to bias in noise on the CS and could be harmful to error correction.

There are various ways to characterize the noise from the environment. For example, dynamical decoupling using filter functions can measure the power spectral density [14,15,16,17]. This has also been measured by observing the relaxation rate in the rotating frame ($T_{1\rho }$) [18]. These methods typically rely on a stochastic description of the noise and take the Markov–Gauss approximation of the environment dynamics. There have been generalizations in measuring the polyspectra of non-Gaussian noise [19,20]. However, these still rely on the truncation of higher-order terms and a classical description of the noise. An experimentally viable method to fully capture the quantum nature of the noise remains elusive.

Previously, M. Niknam et al. [12,21] introduced an experimental method for directly detecting the growth of multi-spin correlations in a CS system and the examination of decoherence time. They defined a metric for quantifying the information flow and entropy distribution in these systems.

This work provides an efficient experimental method, using the stimulated echo (STE) [22,23] and decoupling experiments, for tracking the short- and long-term memory of an environment and for measuring the changes in a local field and in correlation growth. No approximation is taken so that the complete quantum nature of the system is preserved. The memory effect and information flow from the CS to the environment are quantified through autocorrelation functions, which are measured without any truncation. Additionally, the nested commutators between $H_{cs/e}$ and $H_{e}$ are estimated using Bayesian analysis [24,25], which characterizes the growth of memory on different levels.

## 2. Theory

Since our interest is to characterize the buildup of the CS/environment correlations, the initial state of a fiducial state of the CS |0〉〈0| and a fully mixed environment is considered. $H_{cs/e}$ leads to the correlation of the CS and the environment, which is well described by a local field model [26]. The local field is given by the eigenvalues of $H_{cs/e}$ and depends on the environment’s state, which determines the structure of the spectrum of the CS. The growth of correlation under the local field has an initial Gaussian signature and is refocusable by a simple echoing of the CS.

Environmental mixing can alter the state of the environment. For instance, if $H_{e}$ takes the form of a “flip-flop” Hamiltonian $\sigma_{+}⊗\sigma_{-}+\sigma_{-}⊗\sigma_{+}$, then

(2) $$|01\rangle \overset{\sigma_{+}⊗\sigma_{-}+\sigma_{-}⊗\sigma_{+}}{\to }|10\rangle .$$

Such changes in the environment’s state result in a corresponding change in the local field seen by the CS.

The effect of the change in the local field can be described by joint distributions $p(\Omega_{n},\ldots ,\Omega_{1})$, where $\Omega_{i}$ is a random variable representing the local field at time $t_{i}$ (Figure 2), and the Δ’s are the mixing time. The joint distributions can be measured by an *n*-dimensional (*n*D) experiment, which corresponds to the following unitary propagator

(3) $$U_{nD}=e^{-iH_{cs/e}t_{n}}e^{-iH_{e}\Delta_{n-1}}e^{-iH_{cs/e}t_{n-1}}\cdots e^{-iH_{e}\Delta_{1}}e^{-iH_{cs/e}t_{1}}.$$

This separates the effect of the CS/environment interaction and the environment dynamics so that the full dynamics under $H_{cs/e}$ or $H_{e}$ during each time period can be sampled by continuously varying $t_{i}$ or $\Delta_{i}$. A joint distribution $p(\Omega_{n},\ldots ,\Omega_{1})$ can then be obtained by an *n*D Fourier transform with respect to $t_{i}$. In the quantum picture, the variation in the local field corresponds to $H_{cs/e}^{\prime }\left(\Delta \right)=e^{iH_{e}\Delta }H_{cs/e}e^{-iH_{e}\Delta }$, which represents $H_{cs/e}$ in the interaction frame of $H_{e}$. The *n*D experiment effectively compares $H_{s/e}^{\prime }\left(\Delta \right)$ at different times and measures its time dependence, which is caused by the environment dynamics $H_{e}$.

The *n*D experiments provide a more complete picture of the growing correlations, but we do not necessarily need that level of detail. A more efficient approach to measure the memory of the environment is to use the multi-time correlation functions

(4) $$\mathrm{Corr}\left(\Omega_{n},\ldots ,\Omega_{1}\right)=\langle H_{cs/e}^{\prime }\left(\Delta_{n-1}+\cdots +\Delta_{1}\right)\cdots H_{cs/e}^{\prime }\left(0\right)\rangle .$$

Then, it is usually sufficient to implement reduced versions of the *n*D experiments that are typical of lower dimensions [27]. According to the central slice theorem, the spectra of the reduced experiments correspond to projections of the *n*D spectra, which can extract useful information from the spectra.

Here, we show how to measure the autocorrelation function $\langle H_{cs/e}^{\prime }\left(\Delta \right)H_{cs/e}^{\prime }\left(0\right)\rangle$ through the STE experiment. The experiment is a reduced version of the 2D experiment, where $t_{1}$ and $t_{2}$ are set equal and are incremented together. To better understand how the STE experiment works, we can compare the change in the local field to the diffusion in materials (Figure 3).

Indeed, environmental dynamics have been modeled by spin diffusion [28,29], which was inspired by thermal diffusion. A classic experimental method for measuring the thermal diffusion in materials is forced Rayleigh scattering (FRS) [30]. The basic procedure is summarized below.

1. **Creation of spatial grating**: A structured spatial grating is created within the material by intersecting two coherent laser beams.
2. **Thermal diffusion and grating decay**: The material is then allowed to undergo thermal diffusion, which causes the grating to blur.
3. **Monitoring diffusion via scattered light**: A monitoring laser beam (with the same frequency as the initial grating-forming lasers) is applied to the material. The intensity of the scattered light from this probe beam is measured, which indicates the state of the grating. The decay in the scattered intensity corresponds to the extent of diffusion, reflecting the material’s diffusion properties.

The STE experiment (Figure 3) consists of three similar steps.

1. **Creation of the CS/environment correlation**: The CS interacts with the environment via $H_{cs/e}$, forming a correlation with it.
2. **Environment mixing and correlation decay**: The CS undergoes a mixing period under $H_{e}$, which perturbs the CS/environment correlation.
3. **Measuring mixing through echo**: The CS evolves under $-H_{cs/e}$ to form an echo, and the echo intensity is measured to reflect the extent of mixing and reveal properties of the environment.

Here, the correlation plays a similar role to that of the grating, which is used to track the mixing in the environment. Then, the system is allowed to evolve backward under the same Hamiltonian $H_{cs/e}$, similar to the monitoring laser beam, to give a returned signal, which is then used to measure the extent of mixing. By separating the dynamics under $H_{cs/e}$ and $H_{e}$, it allows us to control the extent of dynamics under $H_{cs/e}$ and $H_{e}$ and achieve a better estimation of the parameters in the system.

To understand what information and how it can be extracted from the STE experiment, consider its unitary propagator

(5) $$U_{\mathrm{STE}}=\exp\left(iH_{cs/e}t\right)\exp\left(-iH_{e}\Delta \right)\exp\left(-iH_{cs/e}t\right),$$

and the signal of the experiment is

(6) $$S_{\mathrm{STE}}\left(t,\Delta \right)=\mathrm{Tr}\left(U_{\mathrm{STE}}\rho \left(0\right)U_{\mathrm{STE}}^{†}\rho_{\mathrm{ref}}^{†}\right),$$

where ρ(0) is the density matrix of the initial state of the CS system and $\rho_{\mathrm{ref}}$ is the observable corresponding to a measurement on the CS.

Intuitively, the autocorrelation function $\langle H_{cs/e}^{\prime }\left(\Delta \right)H_{cs/e}^{\prime }\left(0\right)\rangle$ is directly related to the time dependence of $S_{\mathrm{STE}}$ on Δ: the more mixing there is, the less correlated $H_{cs/e}^{\prime }\left(\Delta \right)$ and $H_{cs/e}^{\prime }\left(0\right)$ are, and the weaker the echo is. Indeed, $\langle H_{cs/e}^{\prime }\left(\Delta \right)H_{cs/e}^{\prime }\left(0\right)\rangle$ can be calculated from the second moment (Appendix A) of the spectrum obtained by a Fourier transform of $S_{\mathrm{STE}}\left(t,\Delta \right)$ with respect to *t* (Figure 4). It measures the extent of mixing over any Δ. Fundamentally, the change in the local field arises because $[H_{cs/e},H_{e}]\neq 0$, and this information is encoded in the time dependence of $\langle H_{cs/e}^{\prime }\left(\Delta \right)H_{cs/e}^{\prime }\left(0\right)\rangle$. In fact, the autocorrelation function can be expanded with respect to Δ as

(7) $$\begin{array}{c} \langle H_{cs/e}^{\prime }\left(\Delta \right)H_{cs/e}^{\prime }\left(0\right)\rangle =\\ \;\;\;-\frac{\Delta^{2}}{2!}\langle \left[H_{e},\left[H_{e},H_{cs/e}\right]\right]H_{cs/e}\rangle +\frac{\Delta^{4}}{4!}\langle \left[H_{e},\left[H_{e},\left[H_{e},\left[H_{e},H_{cs/e}\right]\right]\right]\right]H_{cs/e}\rangle +\cdots . \end{array}$$

Only even-order terms appear because $S_{\mathrm{STE}}$ and $\langle H_{cs/e}^{\prime }\left(\Delta \right)H_{cs/e}^{\prime }\left(0\right)\rangle$ are even functions of Δ; changing the sign of Δ does not change $S_{\mathrm{STE}}$. Therefore, the non-commutativity of $H_{cs/e}$ and $H_{e}$ can be measured through estimating the functions $\langle \left[H_{e},\left[H_{e},H_{cs/e}\right]\right]H_{cs/e}\rangle ,\ldots$, providing an absolute measure of mixing that is independent of Δ.

The STE experiment also provides a measure of information flow between the CS and the environment. Note that the decay rate of $S_{\mathrm{STE}}$ with respect to Δ for a fixed *t* depends on the degree of correlation between the CS and the environment: the more correlation there is, the more sensitive the system is to $H_{e}$ and the faster the decay is. Therefore, the decay rate with respect to Δ can be used to monitor the amount of correlation between the CS and the environment. This can also be measured through the spectra obtained by a Fourier transform of $S_{\mathrm{STE}}\left(t,\Delta \right)$ with respect to Δ. The second moment of the spectra is directly related to this sensitivity and gives the autocorrelation function $\langle H_{e}^{\prime }\left(t\right)H_{e}^{\prime }\left(0\right)\rangle$ (Figure 4), where $H_{e}^{\prime }\left(t\right)=\exp\left(iH_{cs/e}t\right)H_{e}\exp\left(-iH_{cs/e}t\right)$ represents $H_{e}$ in the interaction frame of $H_{cs/e}$. $H_{e}^{\prime }\left(t\right)$ can be interpreted as the CS’s perception of the environment. As correlations build, the CS’s view of the environment shifts, causing $\langle H_{e}^{\prime }\left(t\right)H_{e}^{\prime }\left(0\right)\rangle$ to decay.

The time dependence of $\langle H_{e}^{\prime }\left(t\right)H_{e}^{\prime }\left(0\right)\rangle$ can also be used to estimate the non-commutativity of $H_{cs/e}$ and $H_{e}$. Particularly, we can measure terms such as $\langle \left[H_{cs/e},\left[H_{cs/e},H_{e}\right]\right]H_{e}\rangle$ and higher-order nested commutators. These terms provide an absolute measure of the rate of information flow, which are independent of *t*.

Since the STE measures correlation and mixing through the commutation relation between $H_{e}$ and $H_{cs/e}$, it captures only the components that lead to a non-vanishing $\left[H_{e},H_{cs/e}\right]$. There can always be a component with a vanishing commutator that remains unmeasured. For $\langle H_{cs/e}^{\prime }\left(\Delta \right)H_{cs/e}^{\prime }\left(0\right)\rangle$, this unmeasured component corresponds to the part of the environment that does not disrupt the correlation. Conversely, for $\langle H_{e}^{\prime }\left(t\right)H_{e}^{\prime }\left(0\right)\rangle$, it corresponds to the portion of the correlation that remains unaffected by the environment dynamics.

A direct measurement of $H_{cs/e}$ and $H_{e}$ is essential as it establishes a baseline for the intrinsic dynamics of the environment. $H_{cs/e}$ can be determined by analyzing the evolution of the CS under $H_{cs/e}$ while decoupling $H_{e}$, as has been achieved in [12]; a simple spectrum then characterizes the CS/environment coupling. Here, we are mostly interested in measuring $∥H_{e}∥$. For this purpose, the decoupling experiment is employed to assess these dynamics. The experiment explores how the loss of the refocusability of the echo caused by $H_{e}$ changes in the presence of continuous RF irradiation on the environment. The unitary propagator of the experiment is

(8) $$U_{\mathrm{dec}}=\exp\left(-i\left(-H_{cs/e}+H_{e}+\omega_{\mathrm{dec}}J_{x}\right)t\right)\exp\left(-i\left(H_{cs/e}+H_{e}+\omega_{\mathrm{dec}}J_{x}\right)t\right),$$

where $J_{x}$ is the *x* component of the total angular momentum operator of the environment and $\omega_{\mathrm{dec}}$ is the frequency of the RF irradiation. The signal of the experiment is

(9) $$S_{\mathrm{dec}}\left(t\right)=\mathrm{Tr}\left(U_{\mathrm{dec}}\rho \left(0\right)U_{\mathrm{dec}}^{†}\rho_{\mathrm{ref}}^{†}\right).$$

When $\omega_{\mathrm{dec}}$ is small, $H_{e}$ disrupts the CS/environment correlation, leading to a fast decay of $S_{\mathrm{dec}}$ with respect to *t*. When $\omega_{\mathrm{dec}}>∥H_{e}∥$, the RF irradiation becomes dominant, effectively suppressing $H_{e}$ and decoupling the CS from the environment [31]; a rapid slowing in the decay rate can then be observed. Therefore, the frequency $\omega_{\mathrm{dec}}$ at which the transition in the decay rate happens provides a quantitative measure of $∥H_{e}∥$.

## 3. Method

A spin system is used to experimentally demonstrate the method. The experiments are implemented with a high-field (7T) solid-state NMR system at room temperature using a Bruker spectrometer. A powder sample of triphenylphosphine is used to simulate the CS system. The phosphorus spins and the proton spins serve as the CS and environment spins, respectively. The total Hamiltonian of the system is

(10) $$H=\delta_{\mathrm{CS}}\sigma_{z}^{\mathrm{CS}}+\sigma_{z}^{\mathrm{CS}}⊗\sum_{i=1}^{N}\omega_{i}\sigma_{z}^{i}+\sum_{i=1}^{N}\delta_{i}\sigma_{z}^{i}+\sum_{i<j}^{N}\omega_{ij}\left(2\sigma_{z}^{i}⊗\sigma_{z}^{j}-\sigma_{x}^{i}⊗\sigma_{x}^{j}-\sigma_{y}^{i}⊗\sigma_{y}^{j}\right),$$

where *N* is the number of hydrogen spins in the environment; $\delta_{\mathrm{CS}}$ and $\delta_{i}$ are the chemical shift anisotropy of the phosphorus and hydrogen spins, respectively; and $\omega_{i}$ and $\omega_{ij}$ are the coupling strengths of the P-H hetero- and H-H homo-nuclear dipolar interactions, respectively.

**Figure 5:**
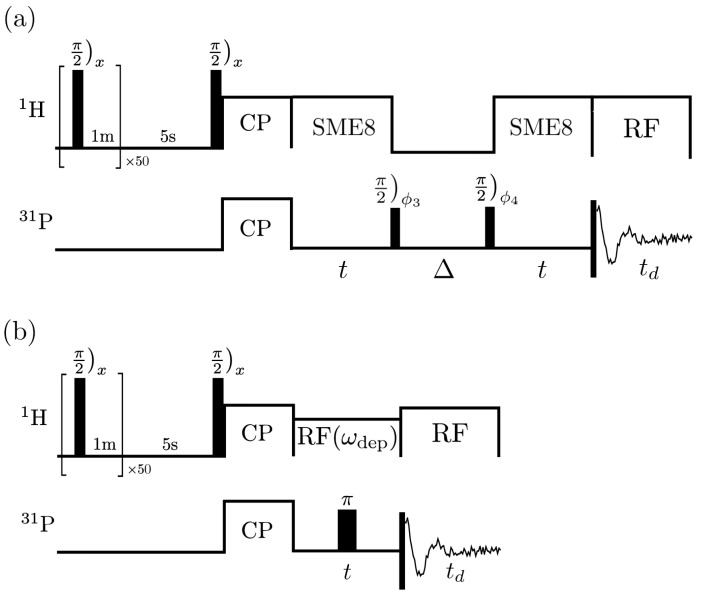
The pulse sequences for the (**a**) STE and the (**b**) decoupling experiments. CP and RF refer to cross-polarization and RF irradiation, respectively. A saturation with π/2 pulses is applied to the environment spins prior to the start of each experiment to prevent correlation between experiments. Spin temperature alteration [38] is incorporated in the phase cycling to remove artifacts from the cross-polarization. During $t_{d}$, a continuous weak measurement is performed on the CS. A strong RF radiation is applied to the environment during this period to decouple the CS from the environment. Then, an integration is taken over $t_{d}$ to give a complex signal point.

**Figure 6:**
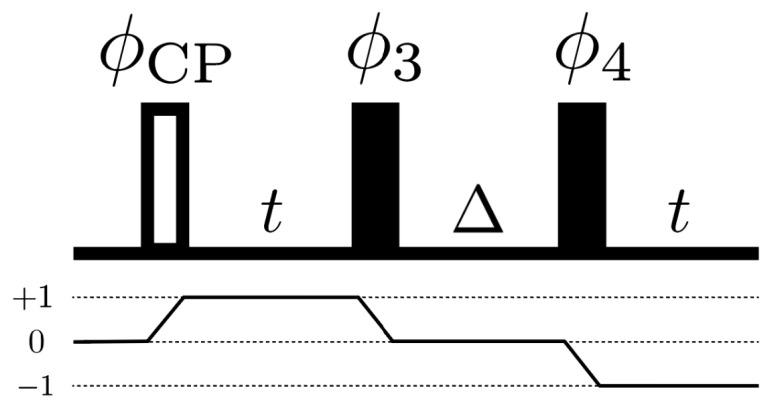
Coherence pathway for STE. The empty rectangle represents the effective excitation pulse corresponding to the cross-polarization. By combining the experiments with carefully chosen phases, one can achieve the desired coherence pathway shown here, which gives the unitary propagator of the STE experiment.

**Figure 1:**
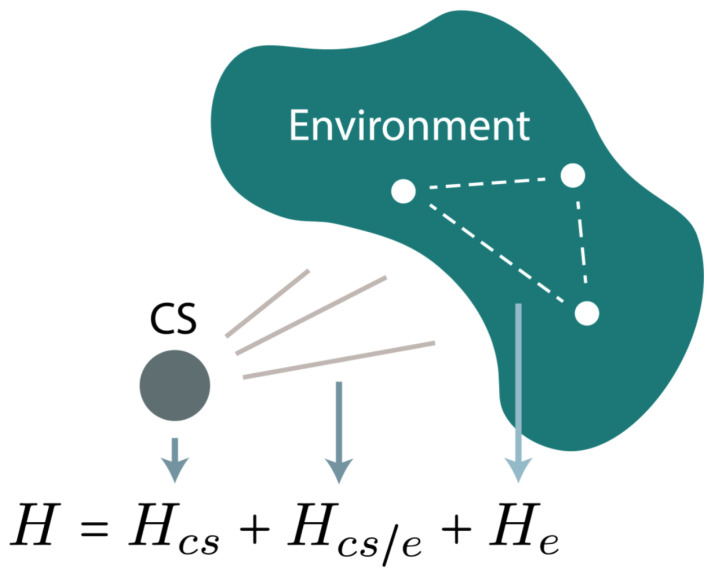
Schematic of a central spin system. The CS is a spin-1/2 particle and interacts with an environment consisting of spin-1/2 particles. The system can provide a complete model of an open quantum system of a qubit.

**Figure 2:**
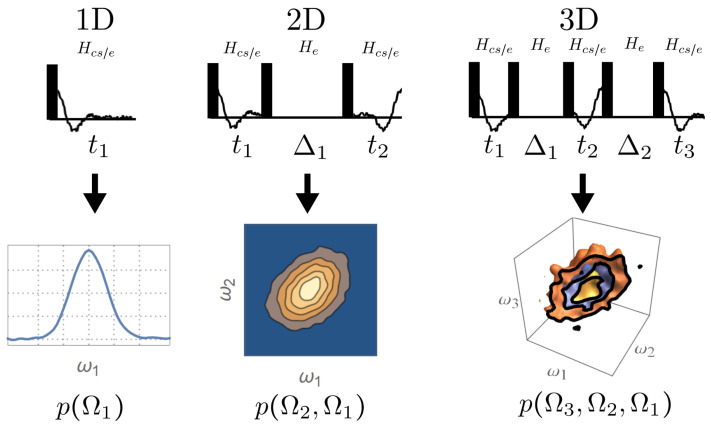
Measuring the multi-time joint distributions of local fields using *n*D experiments. The 1D experiment yields the distribution of local field when there is no mixing. The 2D experiment yields the two-time joint distribution of local fields before and after a period of mixing, which measures the change in the local field. The *n*D experiments (n≥3) can then produce the *n*-time joint distributions that measure the more subtle properties of the memory of the environment.

**Figure 3:**
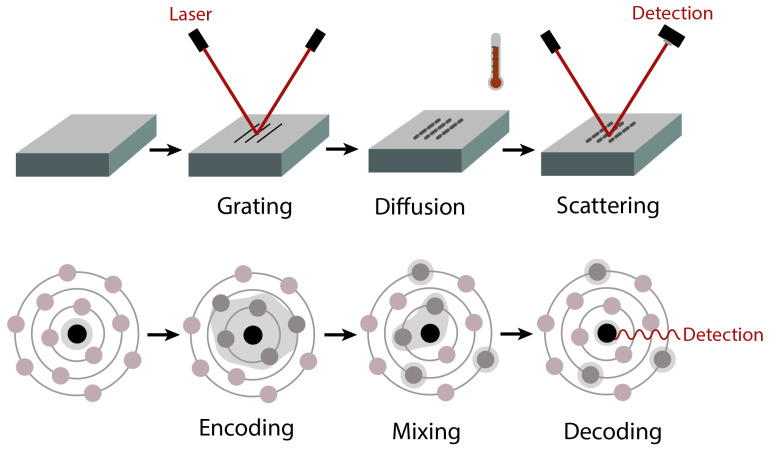
Schematic of the forced Rayleigh scattering (**top**) and stimulated echo (**bottom**) experiments. Like the FRS experiment, the STE experiment consists of three steps (which correspond to grating, diffusion, and scattering, respectively, in the FRS experiment): encoding, mixing, and decoding. The quantum information initially resides at the CS. By comparing it with the echo after decoding, one can learn about mixing.

**Figure 4:**
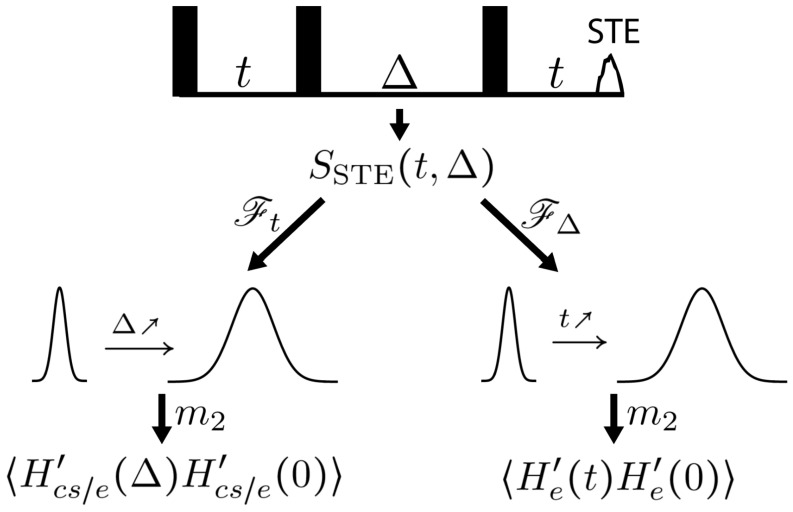
The second moments of the spectra of the STE experiments by Fourier transform with respect to either *t* and Δ give the autocorrelation functions $\langle H_{cs/e}^{\prime }\left(\Delta \right)H_{cs/e}^{\prime }\left(0\right)\rangle$ and $\langle H_{e}^{\prime }\left(t\right)H_{e}^{\prime }\left(0\right)\rangle$, allowing efficient measurement of change in the local field and correlation growth.

The pulse sequences of the STE and decoupling experiments are shown in Figure 5. Correct phase cycling of the π/2 pulses in the STE experiment is chosen to select the desired coherence pathway [32,33] (Figure 6). Additionally, EXORCYLE [34] is applied to the π pulse in the decoupling experiments. Chemical shift anisotropy of the phosphorus spins is refocused by the pulse sequences. That of the proton spins (∼100 Hz) is insignificant and ignored throughout the experiments. The Hamiltonians $H_{cs/e}$ and $H_{e}$ are then given by the P-H heteronuclear dipolar interactions and the H-H homonuclear dipolar interactions, respectively. The sample is doped with a relaxation agent (chromium(iii) acetylacetonate) to reduce its $T_{1}$. The time scale of the experiments (<10 ms) is much smaller than the $T_{1}$ of the phosphorus spins (55 s) and the hydrogen spins (1 s). Cross-polarization [35,36] is used to transfer the polarization from the proton spins to the phosphorus spins to increase the signal-to-noise ratio.

During the encode and decode periods in the STE experiments, the symmetric magic-echo train 8 (SME8) [37] is used to turn off the environment dynamics $H_{e}$ while keeping the system/environment interaction $H_{cs/e}$ with a scaling factor of 0.342 and along the same direction. This, together with the coherence pathway selection, achieves the desired unitary propagator Equation (5).

Bayesian inference was used to estimate the autocorrelation functions and non-commutativity of $H_{cs/e}$ and $H_{e}$ using their corresponding posterior predictive distributions. In addition, a Bayesian model selection [39] (Appendix B) is used to evaluate the goodness of the different levels of models in order to estimate the commutation relations.

## 4. Results

### 4.1. Measuring the Change in the Local Field

**Figure 7:**
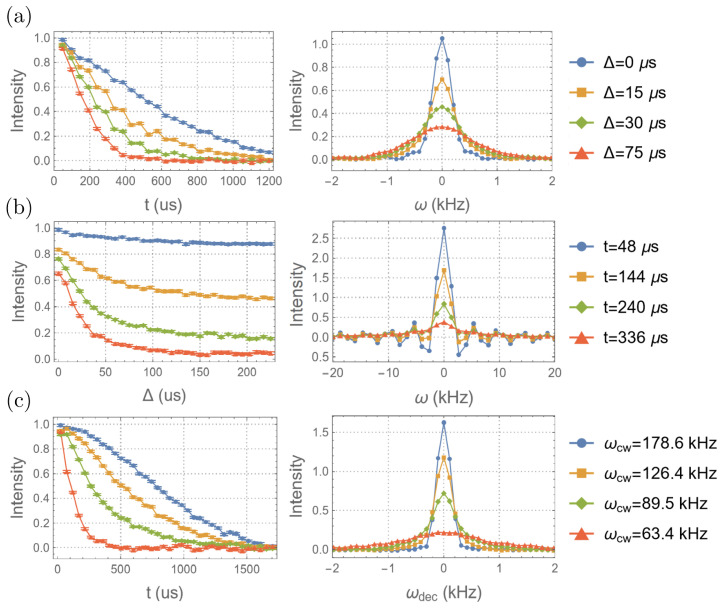
The time-domain data (**left**) and spectra (**right**) of the STE experiments for (**a**) different Δ, (**b**) different *t*, and (**c**) those of the decoupling experiments for different $\omega_{\mathrm{cs}}$. Here, only the real parts of the data are shown. (**a**,**b**) were obtained from the STE experiments (Figure 5a), while (**c**) was obtained from the decoupling experiments (Figure 5b).

STE experiments (Figure 5a) were implemented on the CS system for every combination of t=48,96,…,1200μs, and Δ=0,7.5,…,150μs to collect the signals $S_{\mathrm{STE}}\left(t,\Delta \right)$ (Equation (6)). A Fourier transform on $S_{\mathrm{STE}}$ with respect to *t* yields the spectra ${\tilde{S}}_{\mathrm{STE}}\left(\omega ,\Delta \right)$. The time-domain data $S_{\mathrm{STE}}$ and the corresponding spectra ${\tilde{S}}_{\mathrm{STE}}$ are shown in Figure 7a. As expected, the decay of $S_{\mathrm{STE}}$ with respect to *t* for a given Δ becomes faster as Δ increased, indicating a greater loss of correlation. This is also shown as a broadening of the spectra.

**Figure 8:**
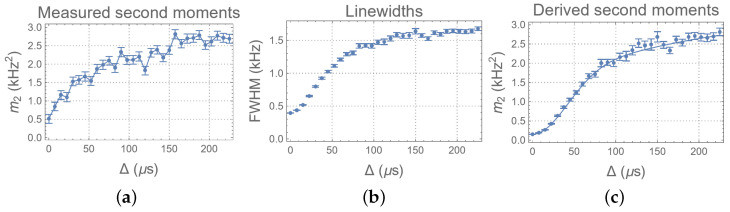
Measure of the (**a**) second moments, (**b**) linewidths, and (**c**) second moments derived from the linewidth of the STE experiments as functions of Δ. The spectra are obtained by Fourier transform with respect to *t*. The data are obtained from their corresponding posterior predictive distributions. The error bars correspond to the 1/4 and 3/4 quantiles. The solid line represents the best fit using the posterior mean for the optimal model with L=2 (Equation (11)).

The second moments of the spectra are calculated to obtain the autocorrelation function $\langle H_{cs/e}^{\prime }\left(\Delta \right)H_{cs/e}^{\prime }\left(0\right)\rangle$. To reduce the uncertainty, the calculation is restricted to the region of the peaks ([−3.6, 3.6] kHz). Note that the frequencies used here are all angular frequencies. The second moments are then evaluated using a Bayesian approach to incorporate the uncertainty (Figure 8a). As seen in the result, the second moment increases (corresponding to the decay of the autocorrelation function) as Δ increases, indicating an increase in the change in the local field. However, due to imperfect refocusing, the signature of a leading-order (second-order) dependence on Δ cannot be observed from the result.

To better explore the features predicted by Equation (7), the linewidths of the spectra are measured and plotted (Figure 8b). The linewidths are measured with less uncertainty and show a clear Gaussian signature for small Δ. Then, the second moments, derived from the measured linewidths by taking a Gaussian approximation ($m_{2}\approx {\mathrm{FWHM}}^{2}/2.355^{2}$), are calculated and plotted in Figure 8c. The derived second moments also show a Gaussian signature, as predicted by Equation (7), and have the same overall timescale as the measured second moments. However, after the initial Gaussian decay, there is a transition (at ∼80 Hz) to a slower decay, suggesting two components of the environment that correspond to different mixing rates.

In order to measure the non-commutativity $\langle \left[H_{e},\left[H_{e},H_{cs/e}\right]\right]H_{cs/e}\rangle ,\ldots$, Bayesian model learning (a combination of model selection and parameter estimation) is applied to the derived second moments according to the model given by Equation (7). To better capture the decay of the autocorrelation function, it is remodeled as a mixture of Gaussians:

(11) $$\langle H_{cs/e}^{\prime }\left(\Delta \right)H_{cs/e}^{\prime }\left(0\right)\rangle =\sum_{l=1}^{L}p_{l}\exp\left(-\Delta^{2}/\lambda_{l}\right),$$

which is equivalent to Equation (7) as L→∞. The procedure for estimating the parameters is as follows: first, the most probable level of the model *L* is selected according to the information criteria calculated by maximizing the likelihood functions; then, a Bayesian analysis is applied to the selected model to obtain the posterior distributions of the parameters $p_{l},\lambda_{l}$; and, finally, the posterior predictive distributions of the functions $\langle \left[H_{e},\left[H_{e},H_{cs/e}\right]\right]H_{cs/e}\rangle ,\ldots$ are obtained from the aforementioned posterior distributions to give the desired estimations.

**Figure 9:**
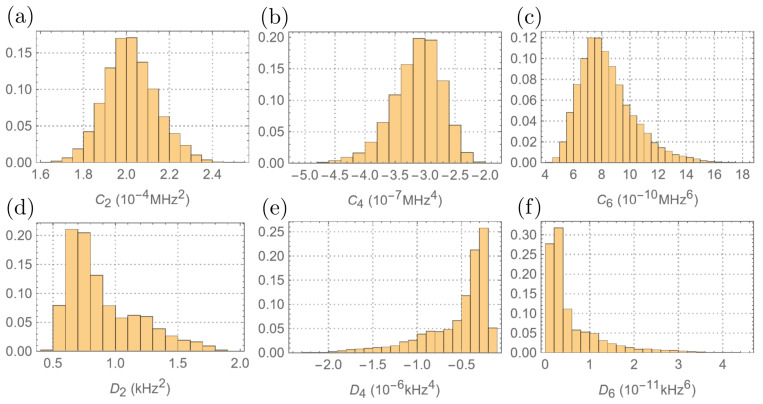
Probability histogram plots of the posterior distributions of (**a**) $C_{2}=\langle \left[H_{e},\left[H_{e},H_{cs/e}\right]\right]H_{cs/e}\rangle$, (**b**) $C_{4}=\langle \left[H_{e},\left[H_{e},\left[H_{e},\left[H_{e},H_{cs/e}\right]\right]\right]\right]H_{cs/e}\rangle$, (**c**) $C_{6}=\langle \left[H_{e},\left[H_{e},\left[H_{e},\left[H_{e},\left[H_{e},\left[H_{e},H_{cs/e}\right]\right]\right]\right]\right]\right]H_{cs/e}\rangle$, (**d**) $D_{2}=\langle \left[H_{cs/e},\left[H_{cs/e},H_{e}\right]\right]H_{e}\rangle$, (**e**) $D_{4}=\langle \left[H_{cs/e},\left[H_{cs/e},\left[H_{cs/e},\left[H_{cs/e},H_{e}\right]\right]\right]\right]H_{e}\rangle$, and (**f**) $D_{6}=\langle \left[H_{cs/e},\left[H_{cs/e},\left[H_{cs/e},\left[H_{cs/e},\left[H_{cs/e},\left[H_{cs/e},H_{e}\right]\right]\right]\right]\right]\right]H_{e}\rangle$.

The selected model is of L=2, confirming the conjecture that there are likely two different components of the environment. The obtained posterior predictive distributions of the first three terms in $\langle \left[H_{e},\left[H_{e},H_{cs/e}\right]\right]H_{cs/e}\rangle ,\ldots$ are plotted in Figure 9 (top row).

The corresponding estimations are summarized in Table 1. The estimations give an absolute measure of the effect of mixing seen by the CS. Higher-order nested commutators are estimated with increased uncertainty. This can be improved by collecting more data for large Δ since the higher-order effects are more important at the tail.

### 4.2. Measuring the Change in the Sensitivity

A similar analysis is applied to the STE experiment data to quantify the information flow. Figure 7b presents the time-domain data and spectra where the roles of Δ and *t* are exchanged. The data show an increased decay rate (spectral broadening) for a larger *t*, reflecting enhanced sensitivity to environmental dynamics due to stronger CS/environment correlations.

**Figure 10:**
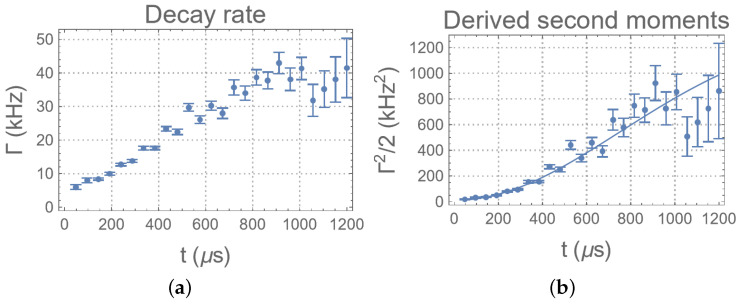
Measurements of the (**a**) linewidths and the (**b**) second moments derived from the linewidths of the STE experiments as functions of *t*. These correspond to the spectra obtained by a Fourier transform of $S_{\mathrm{STE}}$ with respect to Δ.

The linewidths of the spectra are distorted by the Gibbs ringing due to truncation of the time-domain data. Therefore, instead of relying on the direct measurement of the linewidths, the decay rates are estimated by fitting the time-domain data to an exponential function. Then, the decay rate is used to estimate the corresponding second moments (Figure 10). The optimal model, as determined by the Bayesian model selection, has L=1. Then, the commutation relations are estimated by fitting the data to the model. The posterior distributions are shown in Figure 9 (bottom row), with estimates provided in Table 1. Due to the high uncertainty of the data at the tail (t>700μs), the estimates of higher-order commutation relations are significantly worse.

### 4.3. Measuring the Strength of $H_{e}$

**Figure 11:**
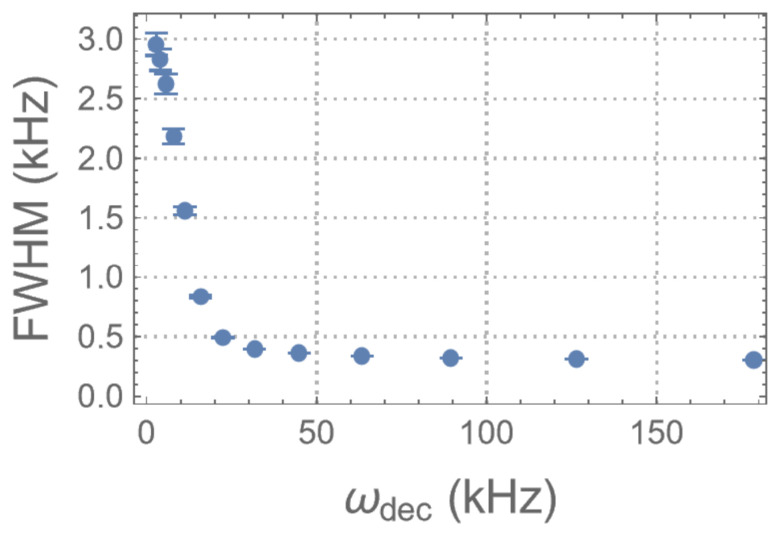
Linewidths of the spectra measured from the decoupling experiments as a function of the strength $\omega_{\mathrm{dec}}$ of the RF irradiation.

To measure the absolute strength of the mixing $∥H_{e}∥$, the decoupling experiments (Figure 5b) are implemented on the CS system for t=48,96,…,1200μs and for different strengths of the RF irradiation. The time-domain data and the corresponding spectra are plotted in Figure 7c. The linewidths of the spectra are calculated and plotted in Figure 11 as a function of $\omega_{\mathrm{dec}}$. The results show that the decay slows as $\omega_{\mathrm{dec}}$ increases, implying that a larger portion of the mixing is suppressed. The Gaussian signature at small $\omega_{\mathrm{dec}}$ corresponds to the regime where $\omega_{\mathrm{dec}}$ becomes larger than $∥H_{cs/e}∥$ and the information flow is being suppressed. It can be seen that the linewidth starts to saturate at around $\omega_{\mathrm{dec}}=$ 30 kHz, indicating the condition that $\omega_{\mathrm{dec}}\gg ∥H_{e}∥$ is satisfied for the majority of the environment. Indeed, the strongest interaction between the environment spins, which are calculated according to the crystal structure of the molecule [40], is around 30 kHz.

## 5. Discussion

The measured autocorrelation function $\langle H_{cs/e}^{\prime }\left(\Delta \right)H_{cs/e}^{\prime }\left(0\right)\rangle$ reveals an overall characteristic time *T*∼90 μs for environment mixing (Figure 8a). However, its structure deviates from an exponential form, indicating memory effects in the environment. Initially, a Zeno regime Δ∼[0,30)μs is observed, corresponding to an initial resistance to the environmental perturbation due to the unitary dynamics of the environment. The duration of the period is approximately equal to $1/∥H_{e}∥$, as measured by the decoupling experiment. Then, this period is followed by a rapid change in the local field, which is characterized by a Gaussian signature, suggesting that the information is being rapidly scrambled in the environment. The environment size is sufficiently large to ensure that this scrambling, as seen by the CS, appears uncorrelated. Around Δ∼70 μs, a transition to a slower change in the local field occurs. Model selection further confirms the presence of two Gaussian components: one fast and one slow. This indicates the existence of a long-lived state within the system/environment correlation that is less sensitive to environmental dynamics. The rapid mixing corresponds to dynamics in a finite-sized part of the environment. Once mixing in this region saturates, the information flow extends to a larger part of the environment, leading to additional information loss. However, this extended part contributes more slowly, likely due to weaker connectivity with the primary environment. This observation is further confirmed through the estimation of non-commutativity, suggesting a structured environment with both long and short memory. A detailed understanding of this memory structure will undoubtedly be crucial for comprehending the environment’s dynamics and for designing improved control and error correction protocols for the system.

Mathematically, the information flow in a many-body system can be quantified by the information flux [41,42]. For the information flow between the CS and the environment, it is closely related to the correlation measure given by the Rényi entropies [21]. The autocorrelation function $\langle H_{e}^{\prime }\left(t\right)H_{e}^{\prime }\left(0\right)\rangle$ and the nested commutation relations $D_{2},D_{4},\ldots$ aim to provide an efficient measure to this correlation. As the results show, the sensitivity to $H_{e}$, as measured by the second moments of the spectra with respect to Δ in STE experiments, reveals a Zeno regime around *t*∼[0,250μs). Similarly, the non-exponential dynamics of the autocorrelation function shows that the CS/environment interaction is highly non-Markovian. According to Niknam et al. [12], first-order coherence peaks around *t*∼200 μs (adjusted for the scaling differences between the MREV8 and SME8 sequences), suggesting that a rapid increase in sensitivity to perturbation mainly arises from higher-order coherence terms. Their results show that coherence growth saturates at *t*∼590 μs. However, in our measurements, sensitivity continues to increase beyond this time. This may imply that highly sensitive, multi-spin correlations continue to develop, even if this does not directly enhance coherence, as most multi-spin correlations are still of lower-order coherence. Thus, STE experiments may be capable of revealing the growth of higher-order correlations.

## 6. Conclusions

In conclusion, an efficient experimental approach combining stimulated echo (STE) and decoupling experiments is presented. It provides quantitative measures of information flow and mixing in a central spin system. The STE experiment distinguishes the effects of system/environment interaction from environment mixing, allowing focused study on either information flow or mixing. The second moments of STE spectra yield autocorrelation functions that capture changes in local fields and sensitivity, offering detailed quantitative insights into the landscape of information flow and mixing. Through Bayesian analysis, the STE experiment directly measures the non-commutativity between $H_{cs/e}$ and $H_{e}$, providing absolute measures of the rates of correlation growth and mixing across different levels. The decoupling experiment, in turn, offers a straightforward and effective method to directly probe the strength of $H_{e}$. Together, the STE and decoupling experiments deliver a comprehensive metric of environment mixing. The results reveal a complex structure in the environment dynamics, with both short-term and long-term memory effects in the system, highlighting the rich dynamics of the central spin system and the importance of thoroughly understanding them. While the method is demonstrated using a spin system, it is applicable to any qubit–environment interacting system.

## Figures and Tables

**Table 1:** Posterior mean estimations of the commutation functions (defined in the caption of Figure 9). The 95% intervals were used to obtain the error bars.

$C_{2}$	$C_{4}$	$C_{6}$
$2.02_{-0.22}^{+0.25}\times 10^{-4}\left({\mathrm{MHz}}^{2}\right)$	$-3.1_{-0.9}^{+0.7}\times 10^{-7}\left({\mathrm{MHz}}^{4}\right)$	$8.4_{-2.9}^{+5}\times 10^{-10}\left({\mathrm{MHz}}^{6}\right)$
$D_{2}$	$D_{4}$	$D_{6}$
$0.9_{-0.35}^{+0.7}\left({\mathrm{kHz}}^{2}\right)$	$-5.3_{-10}^{+3.5}\times 10^{-6}\left({\mathrm{kHz}}^{4}\right)$	$0.6_{-0.5}^{+2}\times 10^{-10}\left({\mathrm{kHz}}^{6}\right)$

**Figure A1:**
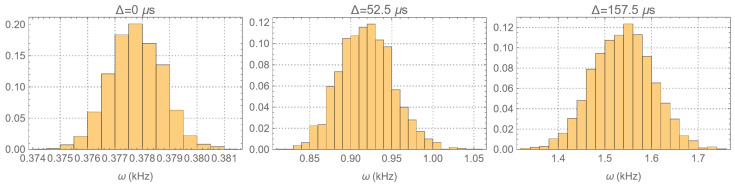
Posterior distributions of the derived second moments from the measure linewidths of the spectra. They are well approximated by Gaussian distributions.

**Figure A2:**
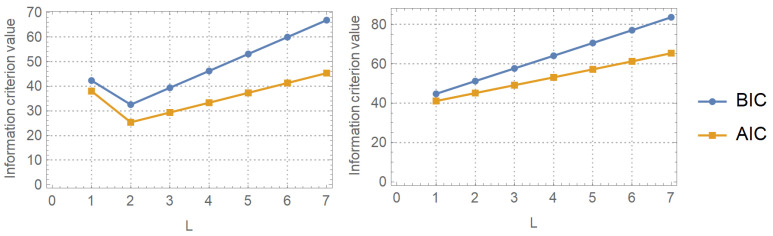
Information criterion values for the models of different order *L* for $\langle H_{cs/e}^{\prime }\left(\Delta \right)H_{cs/e}^{\prime }\left(0\right)\rangle$ (**left**) and $\langle H_{e}^{\prime }\left(t\right)H_{e}^{\prime }\left(0\right)\rangle$ (**right**).

## Data Availability

The original data presented in this study are openly available in Zenodo at https://doi.org/10.5281/zenodo.14183172 (accessed on 18 November 2024).

## Appendix A. Detailed Analysis of the Stimulated Echo

The following Hamiltonians of the system are considered:

(A1) $$H_{cs/e}=\sigma_{z}⊗A/2,\;\;\;H_{e}=1⊗B,$$

where *A* and *B* are operators acting on the environment. The CS/environment interaction is axially symmetric about the *z* direction. The initial state of the system is $\rho \left(0\right)=\sigma_{x}⊗1^{⊗N}$, where *N* is the number of spins in the environment. The identity part of the density matrix is excluded as it does not contribute to the signal. This state represents a scenario where the quantum information initially resides at the CS.

The unitary propagator of the experiment in the interaction frame of $H_{e}$ is

(A2) $$U_{\mathrm{STE}}=\exp\left(iH_{cs/e}^{\prime }\left(\Delta \right)t\right)\exp\left(-iH_{cs/e}^{\prime }\left(0\right)t\right).$$

Therefore, the signal is

(A3) $$S_{\mathrm{STE}}=\mathrm{Tr}\left(e^{-iH_{cs/e}^{\prime }\left(0\right)t}\rho \left(0\right)e^{iH_{cs/e}^{\prime }\left(0\right)t}e^{-iH_{cs/e}^{\prime }\left(\Delta \right)t}\rho_{\mathrm{ref}}^{†}e^{iH_{cs/e}^{\prime }\left(\Delta \right)t}\right),$$

where $\rho_{\mathrm{ref}}=\sigma_{-}⊗1^{⊗N}/2^{N+1}$ is the observable corresponding to a measurement on the CS. When substituting in $H_{cs/e}^{\prime }\left(\Delta \right)=\sigma_{z}⊗A^{\prime }\left(\Delta \right)/2$, where $A^{\prime }\left(\Delta \right)=\exp\left(iB\Delta \right)A\exp\left(-iB\Delta \right)$, and expanding with respect to *t*, we obtain

(A4) $$\begin{array}{cc} \; & \exp\left(-iH_{cs/e}^{\prime }\left(0\right)t\right)\rho \left(0\right)\exp\left(iH_{cs/e}^{\prime }\left(0\right)t\right)=\sigma_{x}⊗\cos\left(A^{\prime }\left(0\right)t\right)+\sigma_{y}⊗\sin\left(A^{\prime }\left(0\right)t\right),\\ \; & \exp\left(-iH_{cs/e}^{\prime }\left(\Delta \right)t\right)\rho_{\mathrm{ref}}\exp\left(iH_{cs/e}^{\prime }\left(\Delta \right)t\right)=\sigma_{+}⊗\exp\left(-iA^{\prime }\left(\Delta \right)t\right)/2^{N+1}. \end{array}$$

Thus, the signal becomes

(A5) $$\begin{array}{cc} S_{\mathrm{STE}} & =\mathrm{Tr}\left(\cos\left(A^{\prime }\left(0\right)t\right)\exp\left(-iA^{\prime }\left(\Delta \right)t\right)+i\sin\left(A^{\prime }\left(0\right)t\right)\exp\left(-iA^{\prime }\left(\Delta \right)t\right)\right)/2^{N}\\ \; & =\mathrm{Tr}\left(\exp\left(iA^{\prime }\left(0\right)t\right)\exp\left(-iA^{\prime }\left(\Delta \right)t\right)\right)/2^{N}. \end{array}$$

Note that $S_{\mathrm{STE}}$ is invariant under the reversal of *t*; thus, it is convenient to write

(A6) $$S_{\mathrm{STE}}=\langle \exp\left(iA^{\prime }\left(\Delta \right)t\right)\exp\left(-iA^{\prime }\left(0\right)t\right)\rangle ,$$

which ensures consistency with the experiment’s time order. The normalization constant is absorbed in the average $\langle \cdot \rangle =\mathrm{Tr}\left(\cdot \right)/2^{N}$ so that the signal is normalized to 1 at Δ=0.

The spectrum ${\tilde{S}}_{\mathrm{STE}}$ is defined as the Fourier transform of $S_{\mathrm{STE}}$ with respect to t:

(A7) $${\tilde{S}}_{\mathrm{STE}}\left(\omega \right)=\frac{1}{\sqrt{2\pi }}\int e^{i\omega t}S_{\mathrm{STE}}\left(t\right)dt.$$

Since

(A8) $$\frac{1}{\sqrt{2\pi }}\int \omega^{n}e^{i\omega t}={\left(-i\right)}^{n}\sqrt{2\pi }\delta^{\left(n\right)}\left(t\right),$$

and

(A9) $$\int f\left(t\right)\delta^{\left(n\right)}\left(t\right)dt=\frac{d^{n}f}{dt^{n}}\left(0\right),$$

we have

(A10) $$m_{k}\left({\tilde{S}}_{\mathrm{STE}}\right)\equiv \int d\omega \omega^{k}{\tilde{S}}_{\mathrm{STE}}=\sqrt{2\pi }{\left(-i\right)}^{k}\frac{d^{k}S_{\mathrm{STE}}}{dt^{k}}\left(0\right).$$

Thus,

(A11) $$\begin{array}{cc} m_{2}\left({\tilde{S}}_{\mathrm{STE}}\right) & =\langle A^{\prime }\left(\Delta \right)A^{\prime }\left(\Delta \right)-A^{\prime }\left(\Delta \right)A^{\prime }\left(0\right)-A^{\prime }\left(0\right)A^{\prime }\left(\Delta \right)-A^{\prime }\left(0\right)A^{\prime }\left(0\right)\rangle \\ \; & =2\langle A^{2}\rangle -2\langle A^{\prime }\left(\Delta \right)A^{\prime }\left(0\right)\rangle . \end{array}$$

Thus, the second moment gives a direct measurement to the autocorrelation function $\langle A^{\prime }\left(\Delta \right)A^{\prime }\left(0\right)\rangle$, which measures the change in the local field for a given Δ.

Expanding the autocorrelation function with respect to Δ, we obtain

(A12) $$\langle A^{\prime }\left(\Delta \right)A^{\prime }\left(0\right)\rangle =\langle A^{2}\rangle +i\Delta \langle \left[B,A\right]A\rangle -\frac{\Delta^{2}}{2}\langle \left[B,\left[B,A\right]\right]A\rangle +\cdots .$$

It is easy to check that all the odd-order terms vanish, which can also be confirmed by noting that $S_{\mathrm{STE}}$ is an even function of Δ. Therefore, the expansion simplifies to

(A13) $$\langle A^{\prime }\left(\Delta \right)A^{\prime }\left(0\right)\rangle =\langle A^{2}\rangle -\frac{\Delta^{2}}{2}\langle \left[B,\left[B,A\right]\right]A\rangle +\frac{\Delta^{4}}{4!}\langle \left[B,\left[B,\left[B,\left[B,A\right]\right]\right]\right]A\rangle +\cdots .$$

The non-commutativity between *A* and *B* (between $H_{cs/e}$ and $H_{e}$) can be obtained by analyzing the time dependence of the second moment of ${\tilde{S}}_{\mathrm{STE}}$.

Note that the signal of the experiment can also be written as

(A14) $$\begin{array}{cc} S_{\mathrm{STE}} & =\langle \exp\left(-iA^{\prime }\left(\Delta \right)t\right)\exp\left(iA^{\prime }\left(0\right)t\right)\rangle \\ \; & =\langle \exp(iB\Delta )\exp(-iAt)\exp(-iB\Delta )\exp(iAt)\rangle \\ \; & =\langle \exp\left(iB^{\prime }\left(t\right)\Delta \right)\exp\left(-iB^{\prime }\left(0\right)\Delta \right)\rangle , \end{array}$$

where $B^{\prime }\left(t\right)=\exp\left(iAt\right)B\exp\left(-iAt\right)$. Denoting by ${\tilde{S}}_{\mathrm{STE}}^{\prime }$ the spectrum obtained by taking Fourier transform of $S_{\mathrm{STE}}$ with respect to Δ, the second moment of the spectrum is

(A15) $$m_{2}\left({\tilde{S}}_{\mathrm{STE}}^{\prime }\right)=2\langle B^{2}\rangle -2\langle B^{\prime }\left(t\right)B^{\prime }\left(0\right)\rangle .$$

The auto-correlation function can be similarly expanded as

(A16) $$\langle B^{\prime }\left(t\right)B^{\prime }\left(0\right)\rangle =\langle B^{2}\rangle -\frac{t^{2}}{2}\langle \left[A,\left[A,B\right]\right]B\rangle +\frac{t^{4}}{4!}\langle \left[A,\left[A,\left[A,\left[A,B\right]\right]\right]\right]B\rangle +\cdots .$$

## Appendix B. Bayesian Analysis for the Stimulated Echo

Bayesian analysis is used to extract information from the experiment data, and the essential steps of the procedure are described here. Each point $x_{i}$ in the time-domain data takes the form

(A17) $$x_{i}=y_{i}+\epsilon_{i},$$

where $y_{i}$ is the corresponding true value and $\epsilon_{i}$ is a zero mean white noise $∼N(0,\sigma^{2})$. The variance $\sigma^{2}$ can be evaluated accurately prehandedly, so it is not included in the parameters to be estimated. Therefore, the likelihood function $p(x_{i}|y_{i})$ for each $x_{i}$ is $N(y_{i},\sigma^{2})$. If we choose the improper prior $p(x_{i})=1$, then the likelihood function is also the posterior distribution $p(y_{i}|x_{i})$.

The posterior predictive distributions of functions *f* of $y_{i}$ can be simulated with samples from $p(\left\{y_{i}\right\}|\left\{x_{i}\right\})$, such as the second moments derived from linewidths or decay rates. Once the posterior predictive distributions are obtained, an estimate is given by taking their means (Bayes estimator).

According to Equation (A11), the *L*th-order model $M_{L}$ of the second moment is given by Equation (11):

(A18) $$M_{L}\left(\Delta ,\vec{a}\right)=C+\sum_{l=1}^{L}p_{l}\exp\left(-\Delta^{2}/\lambda_{l}\right),$$

where $\vec{a}={\left(C,p_{1},\lambda_{1},\ldots ,p_{L},\lambda_{L}\right)}^{T}$ are the parameters to be estimated. The constant *C* absorbs the initial linewidth of the spectra and the possible constant of motion so that the focus is on the time-dependence of the model. The likelihood function $p(\left\{x_{i}\right\}|\vec{a})$ can be connected to the second moments of different Δ through

(A19) $$p\left(\left\{x_{i}\right\}|\vec{a}\right)=\int d\left\{m_{2}\right\}p\left(\left\{x_{i}\right\}|\left\{m_{2}\right\}\right)p\left(\left\{m_{2}\right\}|\vec{a}\right),$$

where $\left\{m_{2}\right\}$ is the collection of second moments for different Δ. Then, $p(\left\{m_{2}\right\}|\vec{a})$ is given by the model $M_{L}$:

(A20) $$p\left(\left\{m_{2}\right\}|\vec{a}\right)=\prod_{\Delta }\delta \left(m_{2}\left(\Delta \right)-M_{L}\left(\Delta ,\vec{a}\right)\right),$$

where δ(·) is the Dirac delta function. The density $p(\left\{x_{i}\right\}|\left\{m_{2}\right\})$ can be evaluated using the Bayes rule:

(A21) $$p\left(\left\{x_{i}\right\}|\left\{m_{2}\right\}\right)=\frac{p\left(\left\{m_{2}\right\}|\left\{x_{i}\right\}\right)p\left(\left\{x_{i}\right\}\right)}{p(\left\{m_{2}\right\})}.$$

Here, $p(\left\{m_{2}\right\}|\left\{x_{i}\right\})$ is the posterior predictive distributions of $m_{2}$, and $p(\left\{x_{i}\right\})$ is a constant independent of $\vec{a}$. If we choose the improper noninformative prior $p(\left\{m_{2}\right\})=1$, then we have $p\left(\left\{x_{i}\right\}|\left\{m_{2}\right\}\right)=p\left(\left\{m_{2}\right\}|\left\{x_{i}\right\}\right)$. Substituting this into the integral, the likelihood function becomes

(A22) $$p\left(\left\{x_{i}\right\}|\vec{a}\right)=\prod_{\Delta }p\left(m_{2}\left(\Delta \right)=M_{L}\left(\Delta ,\vec{a}\right)|\left\{x_{i}\right\}\right).$$

We use the Bayesian information criterion (BIC) [43,44] to evaluate the goodness of each $M_{L}$. It is an approximation of the Bayes factor [45] that is widely used in null hypothesis testing, and defined as

(A23) $$\mathrm{BIC}=n_{\mathrm{par}}\ln\left(n_{\mathrm{data}}\right)-2\ln\left(\hat{L}\right),$$

where $n_{\mathrm{par}}$ is the number of parameters in the model, $n_{\mathrm{data}}$ is the number of data points, and $\hat{L}$ is the maximum likelihood. The first term serves as an Occam’s razor that penalizes the complexity of the model, while the second term evaluates how well the model fits the data. As a comparison, we also calculate the Akaike information criterion (AIC) [46], which is defined as

(A24) $$\mathrm{AIC}=2n_{\mathrm{par}}-2\ln\left(\hat{L}\right).$$

The AIC is more forgiving to a model’s complexity and tries to find the model that best fits the data without overfitting. The BIC tries to find the most plausible underlying model given the available evidence, which is more suitable for our need here.

Instead of using the raw data $\left\{x_{i}\right\}$, we calculate the likelihood function $p(\vec{a}|\left\{m_{2}\right\})$. For convenience of optimization, $p(m_{2}|\left\{x_{i}\right\})$ are approximated by Gaussian distributions with the same mean and variance, which is justified by simulated shapes of the $p(m_{2}|\left\{x_{i}\right\})$ (Figure A1). A generalized simulated annealing optimizer [47,48] is employed to find the maximum likelihood for each model. Parameter ranges are selected to ensure they are wide enough to encompass any reasonable parameter values. The corresponding information criterion values, based on the maximum likelihood identified in the optimization, are plotted in Figure A2. The results show that both the BIC and AIC favored the model with L=2 for $\langle H_{cs/e}^{\prime }\left(\Delta \right)H_{cs/e}^{\prime }\left(0\right)\rangle$ and L=1 for $\langle H_{e}^{\prime }\left(t\right)H_{e}^{\prime }\left(0\right)\rangle$.

The posterior distribution of the parameters is given by

(A25) $$p\left(\vec{a}|\left\{m_{2}\right\}\right)\propto p\left(\left\{m_{2}\right\}|\vec{a}\right)p\left(\vec{a}\right),$$

and an adaptive Metropolis–Hastings sampler [49] is used to simulate the distributions. The prior distributions for the parameters in $\vec{a}$ are chosen as independent logit-normal distributions with σ=1 over the same ranges used in the optimization. The ranges are large enough so that the priors were noninformative. Then, the parameters are transformed to (−∞,+∞) through a logit function to achieve better performance with the sampler.

The posterior distributions of the parameters $C,p_{1},\lambda_{1},\ldots ,p_{L},\lambda_{L}$ are obtained for the desired model (L=2), then the non-commutativity is measured using the corresponding posterior predictive distributions through the following relation:

(A26) $$C_{k}\;\mathrm{or}\;D_{k}=-\frac{1}{2}\frac{1}{p_{1}+\cdots +p_{L}}\frac{k!}{(k/2)!}\sum_{l=1}^{L}{\left(-1\right)}^{k/2}\frac{p_{l}}{\lambda_{l}^{k/2}},$$

where $C_{k}=\langle \left[B,\left[B,A\right]\right]A\rangle ,\ldots$ and $D_{k}=\langle \left[A,\left[A,B\right]\right]B\rangle ,\ldots$, for k=2,4,…, being the level of the nested commutator, are the measured commutation relations. Note that the result is normalized with respect to $\langle A^{2}\rangle$ and $\langle B^{2}\rangle$ so that $C_{0}=D_{0}=1$.
